# Viewing the Body after Bereavement Due to Suicide: A Population-Based Survey in Sweden

**DOI:** 10.1371/journal.pone.0101799

**Published:** 2014-07-07

**Authors:** Pernilla Omerov, Gunnar Steineck, Tommy Nyberg, Bo Runeson, Ullakarin Nyberg

**Affiliations:** 1 Centre for Psychiatric Research and Education, Department of Clinical Neuroscience, Karolinska Institutet, Stockholm, Sweden; 2 Division of Clinical Cancer Epidemiology, Department of Oncology and Pathology, Karolinska Institutet, Stockholm, Sweden; 3 Division of Clinical Cancer Epidemiology, Department of Oncology, Institute of Clinical Sciences, The Sahlgrenska Academy, Gothenburg, Sweden; Harvard Medical School, United States of America

## Abstract

**Background:**

Research on the assumed, positive and negative, psychological effects of viewing the body after a suicide loss is sparse. We hypothesized that suicide-bereaved parents that viewed their childs body in a formal setting seldom regretted the experience, and that viewing the body was associated with lower levels of psychological morbidity two to five years after the loss.

**Methods and Findings:**

We identified 915 suicide-bereaved parents by linkage of nationwide population-based registries and collected data by a questionnaire. The outcome measures included the Patient Health Questionnaire (PHQ-9). In total, 666 (73%) parents participated. Of the 460 parents (69%) that viewed the body, 96% answered that they did not regret the experience. The viewing was associated with a higher risk of reliving the child's death through nightmares (RR 1.61, 95% CI 1.13 to 2.32) and intrusive memories (RR 1.20, 95% CI 1.04 to 1.38), but not with anxiety (RR 1.02, 95% CI 0.74 to 1.40) and depression (RR 1.25, 95% CI 0.85 to 1.83). One limitation of our study is that we lack data on the informants' personality and coping strategies.

**Conclusions:**

In this Swedish population-based survey of suicide-bereaved parents, we found that by and large everyone that had viewed their deceased child in a formal setting did not report regretting the viewing when asked two to five years after the loss. Our findings suggest that most bereaved parents are capable of deciding if they want to view the body or not. Officials may assist by giving careful information about the child's appearance and other details concerning the viewing, thus facilitating mental preparation for the bereaved person. This is the first large-scale study on the effects of viewing the body after a suicide and additional studies are needed before clinical recommendations can be made.

## Introduction

Viewing the body after a sudden death is often said to be helpful for bereaved family members [1], [2]. Chapple and Ziebland [1] found that relatives, bereaved through suicide or other traumatic deaths, who had chosen to view the body seldom regretted doing so. They also found that the relatives often had numerous reasons for viewing the body and mentioned the need for checking the identity, to care for the deceased and to say goodbye. These findings were based on 80 in-depth interviews conducted in Great Britain between 2007 and 2008, four months to nine years after the loss. The benefits of viewing the body after an unexpected death may also be explained by applying grief theories and the notion that facing the dead person facilitates the grief process by bringing reality to the death and by providing an opportunity for closures [1], [3], [4]. There are also relatives who do not want to view the body; some want to remember the person as he or she was when being alive, others want to spare themselves from a fearful sight and unwanted memories [1]. The fear of unwanted memories is also an explanation to why health care professionals sometimes are unwilling to show a disfigured body [5]. Research on the assumed (positive and negative) psychological effects of viewing the body after a suicide loss is however sparse.

In this population-based study we used the personal identification numbers and the nationwide high-quality registers to identify a large sample of unselected suicide-bereaved parents in Sweden. We thereafter used a detailed questionnaire with psychometric scales and study-specific questions to test our hypotheses: parents that viewed their childs body in a formal setting seldom regretted the experience, and that viewing the body was associated with lower levels of psychological morbidity two to five years after the loss.

## Methods

### Ethics statement

We identified the study population by linkages of registers. In Sweden, the use of register data always needs ethical approval by the regional ethical review boards. Additionally, the register holders make a risk assessment related to The Law on Public Disclosure and Security. We contacted all parents by means of an introduction letter followed by a telephone call. The letter contained information about the study and contact details for the researchers. In the letter we emphasized that participation was voluntary and informed about the possibility to end participation at any time without further explanation. During the telephone call we repeated the information from the letter and asked if the parent wanted to participate and if we could send a questionnaire. The informed oral consent of participation was noted in our database and confirmed by a returned and completed questionnaire. For ethical reasons, we did not obtain a written consent during contact as we did not want the parents to feel pressured to complete participation. The data used in this paper were analyzed anonymously; we could therefore not obtain a written consent afterwards. Our study as well as our contact and consent procedures was approved by the Regional Ethical Review Board in Stockholm, Sweden. Our ethical protocol for data collection and contact is published at: http://dx.doi.org/10.1017/S0033291713001670 [6].

### Subjects

We identified all individuals, 15 to 30 years old, who died by suicide (ICD 10: X60–X84) between 2004 and 2007 and also identified their parents by linkage of the nationwide Swedish Cause of Death Register and the Multi-generation Register [7]. To be included in the study, the parent had to be born in one of the Nordic countries, be able to communicate in Swedish and have an identifiable address and telephone number. Furthermore, parents who had lost more than one child were excluded. In total, 915 parents were identified as eligible.

### Data collection and measurements

We developed the study design from the routines established by the Division of Clinical Cancer Epidemiology [6], [8]–[10]. Using qualitative methods, we formed study-specific questions on the basis of seventeen in-depth interviews with suicide-bereaved parents [9]. Psychological outcomes were measured by: The two-item Generalized Anxiety Disorder scale (GAD-2) [11], [12] and The nine-item depression scale of the Patient Health Questionnaire (PHQ-9) [13], [14]. We used study specific questions, with space for free comments, to assess circumstances related to the suicide and the viewing (presented in table 1,2). Furthermore, we used four questions with follow-up questions (presented in table 3) to asses if the parents had viewed the body in a formal setting and if it had been during dignified circumstances. To assess the prevalence of nightmares, intrusion and avoidance related to the child's death we used the study specific questions presented in table 4. All questions, including the psychometric scales, were tested in a preparatory study that included 46 suicide-bereaved persons from our study population [9]._ENREF_1 We contacted all eligible parents by an introductory letter followed by a telephone-call to obtain consent to send a questionnaire. Parents of the same child were contacted separately and each individual received a questionnaire of their own. We started the data collection in August 2009 and the last questionnaire was returned in December 2010 [9].

**Table 1:** Sociodemographic characteristics of the suicide-bereaved parents.

	Suicide-bereaved parents	
	Viewed at formal	Did not view at
	setting*	formal setting
Sex – no. (%)		
Fathers	185/282 (65.6)	97/282 (34.4)
Mothers	275/380 (72.4)	105/380 (27.6)
Age – yr		
Fathers, Median (Interquartile range)	58 (54 to 62)	58 (53 to 62)
Mothers, Median (Interquartile range)	55 (51 to 59)	56 (52 to 60)
Year of child's death – no. (%)		
2004	111/162 (68.5)	51/162 (31.5)
2005	114/171 (66.7)	57/171 (33.3)
2006	123/168 (73.2)	45/168 (26.8)
2007	112/161 (69.6)	49/161 (30.4)
Age deceased child – yr Median (Interquartile range)	23 (20 to 26)	24 (20 to 28)
Sex deceased child – no. (%)		
Male	319/458 (69.7)	139/458 (30.3)
Female	141/204 (69.1)	63/204 (30.9)
Children – no. (%)		
No remaining children	27/47 (57.4)	20/47 (42.6)
Remaining children	433/615 (70.4)	182/615 (29.6)
Biological child – no. (%)		
Non biological child	21/31 (67.7)	10/31 (32.3)
Biological child	439/631 (69.6)	192/631 (30.4)
Family constellation at time of study – no. (%)		
Living with a partner	345/475 (72.6)	130/475 (27.4)
Has a partner but lives alone	27/44 (61.4)	17/44 (38.6)
Single	78/121 (64.5)	43/121 (35.5)
Widow, widower	8/18 (44.4)	10/18 (55.6)
Residence area – no. (%)		
Rural	111/161 (69.0)	50/161 (31.0)
Village (population less than 10,000)	111/153 (72.5)	42/153 (27.5)
Small town (population less than 50,000)	87/127 (68.5)	40/127 (31.5)
Town (population less than 200,000)	77/117 (65.8)	40/117 (34.2)
Larger town (population more than 200,000)	71/97 (73.2)	26/97 (26.8)
Country of birth – no. (%)		
Born in Sweden	437/625 (70.0)	188/625 (30.0)
Born in other Nordic country	22/36 (61.1)	14/36 (38.9)
Level of education – no. (%)		
Less than elementary school	1/5 (20.0)	4/5 (80.0)
Elementary school	105/141 (74.5)	36/141 (25.5)
Junior college	179/270 (66.3)	91/270 (33.7)
College or university (< 3 years)	57/82 (69.5)	25/82 (30.5)
College or university (≥ 3 years)	116/159 (73.0)	43/159 (27.0)
Source of income – no. (%)		
Employed or self-employed	350/496 (70.6)	146/496 (29.4)
Old-age pension	36/59 (61.0)	23/59 (39.0)
Disability pension	44/61 (72.1)	17/61 (27.9)
Unemployment fund	19/25 (76.0)	6/25 (24.0)
Other	9/16 (56.2)	7/16 (43.8)
Religion – no. (%)		
Do not believe in God	245/354 (69.2)	109/354 (30.8)
Believes in God	200/286 (69.9)	86/286 (30.1)

^*^ Parents that stated that they viewed their dead child in a formal setting. We asked if they viewed in the body at “The emergency department or ward”, “Hospital church”, “Department of forensic medicine”, and “Funeral parlour”. Viewing also includes viewing the contour of the body or part of the body.

**Table 2:** Circumstances related to the suicide.

	Suicide-bereaved parents	
	Viewed body	Did not view
	no./total no. (%)	no./total no. (%)
How did your child commit suicide		
Poisoning*	64/101 (63.4)	37/101 (36.6)
Hanging, strangulation, suffocation	266/345 (77.1)	79/345 (22.9)
Drowning	3/8 (37.5)	5/8 (62.5)
In front of moving vehicles	37/81 (45.7)	44/81 (54.3)
Jumping from a height	36/46 (78.3)	10/46 (21.7)
By firearm discharge	29/45 (64.4)	16/45 (35.6)
Other way	16/24 (66.6)	8/24 (33.3)
How did you know that your child was deceased		
Found dead child	86/109 (78.9)	23/109 (21.1)
Saw dead child at site but not as first person	23/32 (71.9)	9/32 (28.1)
Notified in person	207/297 (69.7)	90/297 (30.3)
Notified by telephone	108/179 (60.3)	71/179 (39.7)
Other way†	34/42 (80.0)	8/42 (20.0)
Did you receive the death notice from a professional person		
No	201/292 (68.8)	91/292 (31.2)
Yes	251/358 (70.1)	107/358 (29.9)
If yes, did the person come to your home		
No	95/139 (68.3)	44/139 (31.7)
Yes	186/268 (69.4)	82/268 (30.6)
If yes, did the person stay as long as you wanted		
No, too short	32/45 (71.1)	13/45 (28.9)
No, too long	4/5 (80.0)	1/5 (20.0)
Yes	176/257 (68.5)	81/257 (31.5)
Where you informed that your child died by suicide at the time of the death notice		
No	52/68 (76.5)	16/68 (23.5)
Yes	339/508 (66.7)	169/508 (33.3)
Was the death notice given in a dignified way		
No	61/79 (77.2)	18/79 (22.8)
Yes, a little	51/75 (68.0)	24/75 (32.0)
Yes, moderately	78/112 (69.6)	34/112 (30.4)
Yes, much	144/225 (64.0)	81/225 (36.0)
Where you prepared that your child might have committed		
suicide, when you received the death notice		
No	261/361 (72.3)	100/361 (27.7)
Yes, a little	64/88 (72.7)	24/88 (27.3)
Yes, moderately	22/33 (66.7)	11/33 (33.3)
Yes, much	83/138 (60.1)	55/138 (39.9)
How long time proceeded between your child's death and		
you being notified about his or her death		
0 – 3 hours	151/208 (72.6)	57/208 (27.4)
4 – 6 hours	93/131 (71.0)	38/131 (29.0)
7 – 12 hours	97/137 (70.8)	40/137 (29.2)
13 – 23 hours	56/79 (70.9)	23/79 (29.1)
1 – 3 days	47/71 (66.2)	24/71 (33.8)
4 – 6 days	7/19 (36.8)	12/16 (63.2)
1 – 3 weeks	3/7 (42.9)	4/7 (57.1)
One month or more	0/3 (0.0)	3/3 (100.0)

^*^ Poisoning for example by medication, chemicals or some kind of gas”.

† Of the 40 parents that stated “Other way” 17 wrote that they were present at the time of death; 11 at the hospital and 6 had witnessed the suicide, 23 parents wrote that they received the death notice from someone else and two did not comment on the question.

**Table 3:** Suicide-bereaved parents experience of viewing the body at formal settings.

no./tot no. (%)	No	Yes	*a little*	*moderate*	*much*	Missing
**Did you view your child at:**						
**The Hospital (ER, Ward)**	517 (77.6)	140 (21.0)				9 (1.4)
*If yes, was it during*	8 (5.7)		11 (7.9)	22 (15.7)	97 (69.3)	2 (1.4)
*dignified circumstances*						
**The Hospital church**	431 (64.7)	227 (34.1)				8 (1.2)
*If yes, was it during*	7 (3.1)		9 (4.0)	30 (13.2)	178 (78.4)	3 (1.3)
*dignified circumstances*						
**Forensic medicine**	555 (83.3)	98 (14.7)				13 (2.0)
*If yes, was it during*	2 (2.0)		4 (4.1)	15 (15.3)	73 (74.5)	4 (4.1)
*dignified circumstances*						
**The Funeral parlour**	448 (67.3)	209 (31.4)				9 (1.4)
*If yes, was it during*	5 (2.4)		3 (1.4)	17 (8.1)	176 (84.2)	8 (3.8)
*dignified circumstances*						
**Any of the above** *	202 (30.3)	460 (69.1)				4 (0.6)
*If yes, was it during*	19 (4.1)		21 (4.6)	63 (13.7)	352 (76.5)	5 (1.1)
*dignified circumstances* †						

^*^ “Emergency department or ward”, “Hospital church”, “Department of forensic medicine”, and “Funeral parlour”. Viewing also includes viewing the contour of the body or part of the body.

† The most unfavourable value ranging from “No”; “Yes, a little”; “Yes, moderate”; “Yes, much” at any of the formal settings.

**Table 4:** Psychological outcomes among the parents that viewed and did not view the body.

	Suicide-bereaved parents		
Variables no.	Viewed in a	Did not view in a	Trend test
/total no. (%)	formal setting*	formal setting	P value
**Relived child's death through**			
**nightmares the last month** †	114/460 (24.8)	31/202 (15.3)	
Relative Risk (95% CI)	1.61 (1.13 to 2.32)	1.0 (reference)	0.005
Unadjusted odds ratios	1.82 (1.17 to 2.81)	1.0 (reference)	
Adjusted odds ratios‡ § **	1.85 (1.16 to 2.95)	1.0 (reference)	
**Relived child's death through**			
**intrusive memories the last month** †	297/455 (65.3)	109/200 (54.5)	
Relative Risk (95% CI)	1.20 (1.04 to 1.38)	1.0 (reference)	0.007
Unadjusted odds ratios	1.57 (1.12 to 2.20)	1.0 (reference)	
Adjusted odds ratios‡ § ††	1.50 (1.04 to 2.16)	1.0 (reference)	
**Avoided thinking about things that**			
**reminds about child's death the last month** †	156/458 (34.1)	57/200 (28.5)	
Relative Risk (95% CI)	1.20 (0.93 to 1.54)	1.0 (reference)	0.276
Unadjusted odds ratios	1.30 (0.90 to 1.86)	1.0 (reference)	
Adjusted odds ratios‡ § ‡‡	1.28 (0.86 to 1.91)	1.0 (reference)	
**Avoided things that reminds about child's**			
**death the last month e.g. places and things** †	118/457 (25.8)	52/197 (26.4)	
Relative Risk (95% CI)	0.98 (0.74 to 1.30)	1.0 (reference)	0.927
Unadjusted odds ratios	0.97 (0.66 to 1.42)	1.0 (reference)	
Adjusted odds ratios‡ § § §	1.01 (0.66 to 1.54)	1.0 (reference)	
**Depression (PHQ-9 score ≥ 10)** ***	85/452 (18.8)	30/199 (15.1)	
Relative Risk (95% CI)	1.25 (0.85 to 1·83)	1.0 (reference)	0.005
Unadjusted odds ratios	1.30 (0.83 to 2·06)	1.0 (reference)	
Adjusted odds ratios‡ ††† ‡‡‡	1.27 (0.76 to 2.12)	1.0 (reference)	
**Anxiety (GAD-2 score ≥ 2)** § § §	97/454 (21.4)	42/200 (21.0)	
Relative Risk (95% CI)	1.02 (0.74 to 1.40)	1.0 (reference)	0.893
Unadjusted odds ratios	1.02 (0.68 to 1.54)	1.0 (reference)	
Adjusted odds ratios‡ ††† ****	0.89 (0.56 to 1.40)	1.0 (reference)	

^*^ Parents that stated that they viewed their dead child in a formal setting. We asked if they viewed in the body at “The emergency department or ward”, “Hospital church”, “Department of forensic medicine”, and “Funeral parlour”.

† “No”“ Yes, occasionally”, “Yes, 1–3 days a week”, “Yes, 4–5 days a week”, “Yes, 6–7 days a week”. Dichotomized into “No” and “Yes”.

‡ OR adjusted for multiple variables selected by logistic regression forward selection. Variables that met the 0.15 significant level were included in the models.

§ Variables in the selection: sex, age, residence, civil status, income, education. physical activity, social activity, violent suicide, found dead child, death notice, contact, AUDIT, PHQ, GAD, sleeping pill, anxiolytics, and antidepressants.

^**^ Selected variables: GAD, sleeping pill, education, and sex.

†† Selected variables: GAD, sex, sleeping pill, physical activity, and age.

‡‡ Selected variables: PHQ, social activity, sex, GAD, and age.

§ § Selected variables: PHQ, sex, social activity, GAD, physical activity, income and, violent suicide.

^***^ PHQ-9 score 0–27. Answering categories: “Not at all”, “1–3 days a week”,“4–5 days a week”, and “6–7 days a week”.

††† Variables in the selection: sex, age, residence, civil status, income, education. physical activity, social activity, violent suicide, found dead child, death notice, contact, and AUDIT.

‡‡‡ Selected variables: Income, sex, AUDIT, social activity, physical activity, age, and civil status.

§ § § GAD-2 scores 0–6. Answering categories: “Not at all”, “1–3 days a week”,“4–5 days a week”, and “6–7 days a week”.

^****^ Selected variables: Income, sex, physical activity, social activity, AUDIT, and age.

### Statistical analysis

We used Wilcoxon-Mann-Whitney's test to assess the association between viewing the body or not viewing the body and the levels of psychological outcomes. Using recommended cut-offs [11], [13] we dichotomized the scores derived from the psychometric scales and used log-binomial regression to calculate relative risks. We performed a variable selection among possible confounders, using logistic regression with forward selection in order to identify those variables most strongly related to the outcomes. Since we wanted to maximize the possibility to find other explanatory factors that could potentially disprove the assumed effect of viewing the body, we used a liberal inclusion criterion allowing variables up to the 15% significance level entry. We then formed one final model for each outcome utilizing all variables that had been identified as associated with the outcome and reported the results by adjusted odds ratios. As sensitivity analyses we stratified according to whether the parents had had regular contact with the child during the year preceding the suicide, and we also divided the parents into different groups related to whether they: 1) saw the childs body at the site of death 2) only saw the childs body in a formal setting and 3) did not see the childs body at all. We performed statistical tests at the 5% significance level unless otherwise stated and excluded individuals with missing data in each respective calculation. All statistical analyses were performed with IBM SPSS Statistics software, version 19.0.

## Results

### Primary outcomes

The questionnaires were returned by 666 of the 915 (73%) suicide-bereaved parents, 460 (69%) of whom stated that they had viewed the body in a formal setting, 202 (30%) that they had not, and four (<1%) did not respond to the questions (Table 1). The question “Do you regret that you viewed your child after the death” was answered by 456 of the 460 parents that had viewed. Ten answered that they had not viewed the body. Of the remaining 446, 430 (96%) answered “No”, 9 (2%) “Yes, a little”, 2 (<1%) “Yes, moderately” and, 5 (1%) “Yes, much” (Data not shown in table). According to the written comments, several of the parents that regretted viewing the child had witnessed a decomposed body. Some of the ones that regretted viewing also wrote that they wished that they had been better prepared for the scene that met them. Regrets were significantly lower among those who had lost a son or daughter to a violent suicide than among those who had lost a son or daughter by poisoning (relative risk 0.19, 95 percent confidence interval 0.07 to 0.49) (Table S1). Reported regrets related to viewing the body in a formal setting did not differ significantly between those who stated that they had seen the body at the site of death and those who had not, and the results were similar also when stratifying according to whether the parent had had regular contact with the child or not (data not shown in table).

The question “Do you wish that you had viewed your child after the death” was answered by 198 of the 202 parents that did not view the body in a formal setting. Thirty-nine answered that they had viewed the child. Of the remaining 159, 99 (62%) answered “No”, 25 (16%) “Yes, a little”, 11 (7%) “Yes, moderately” and, 24 (15%) “Yes, much” (Data not shown in table). According to the written comments several of the ones that did not view the body had been advised by the officials not to do so, since the body was severely damaged or had started to decompose.

Five of the 460 parents that had viewed the body in a formal setting did not answer any of the questions regarding whether they perceived that the viewing was performed in a dignified way. Of the remaining 455, 19 (4%) answered “No”, 21 (5%) “Yes, a little”, 63 (14%) “Yes, moderately” and, 352 (77%) “Yes, much” on at least one question regarding if the viewing was performed during dignified circumstances (Table 2).

### Secondary outcomes

**Figure 1:**
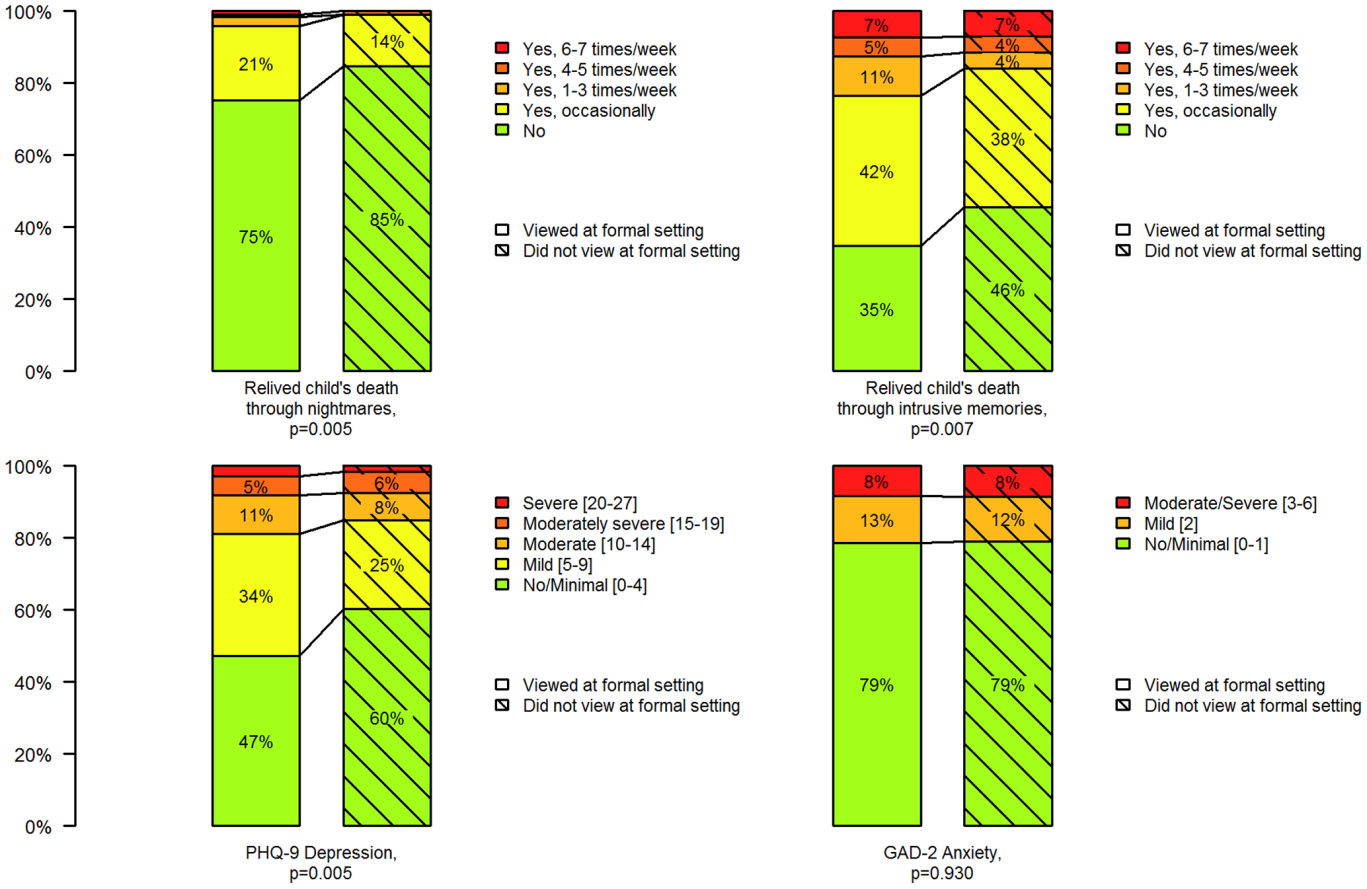
Psychological outcomes among the parents that viewed and did not view the body.

Viewing the child in a formal setting was associated with a statistically significantly higher risk of having relived the child's death through nightmares (relative risk 1.61, 95 percent confidence interval 1.13 to 2.32) and intrusive memories (relative risk 1.20, 95 percent confidence interval 1.04 to 1.38) at least occasionally during the preceding month. No statistically significant difference was found concerning anxiety (GAD-2, score ≥ 2) (relative risk 1.02, 95 percent confidence interval 0.74 to 1.40) and depression (PHQ-9, score ≥ 10) (relative risk 1.25, 95 percent confidence interval 0.85 to 1.83) (Figure 1, Table 4). The psychological morbidity related to viewing the body in a formal setting was found both among those who reported that they had seen the body at the site of death and those who had not (data not shown in table). Not seeing the body at all or only seeing the body at the site of the death (not in a formal setting) was not associated with elevated risks of psychological outcomes 2-5 years after the loss (Table S1).

## Discussion

This is the first large population-based study on psychological reactions to viewing the body after a suicide. We found that by and large everyone of the 460 parents that had viewed their deceased child in a formal setting did not regret the viewing when asked two to five years after the loss. Of equal importance, more than half of the 202 parents who did not view the body did not wish that they had. In contrast to what we hypothesized, we found that those parents who had viewed the body in a formal setting had a statistically significantly higher risk of reliving the child's death through nightmares (relative risk 1.61, 95 percent confidence interval 1.13 to 2.32) and intrusive memories (relative risk 1.20, 95 percent confidence interval 1.04 to 1.38). We found no statistically significant difference concerning anxiety (relative risk 1.02, 95 percent confidence interval 0.74 to 1.40) and depression (relative risk 1.25, 95 percent confidence interval 0.85 to 1.83) (Table 4).

Our findings that most parents who viewed the body do not regret doing so correspond with findings from previous studies [1], [2]. As in Chapple and Ziebland's study [1], only a few persons stated that they regretted viewing of the body. In our study regrets were often followed by a comment that expressed shock over how their loved ones had changed. Providing information on what to expect has been stressed as an important element in reducing distress and regrets due to viewing the body after a traumatic death [1], [2], [15]. Interestingly, in our study, regrets were most often associated with death by poisoning rather than a violent method of suicide. The written comments also showed that the regrets mainly concerned witnessing a decomposed body rather than a body that was disfigured by the suicide. Possible explanations might be that after a violent death the relatives are better informed on what to expect and the body is more often shielded. The violently bereaved parents might also expect the worst. Our findings suggest it is always important to inform the parents about the body's appearance and about options for the viewing, whatever the mode of death. Health care personnel are often encouraged to carefully prepare the environment and the body before the viewing [2], [16], [17]. However, after an as of yet unverified suicide, cleaning the body may be delayed due to an ongoing police investigation. In our study, most parents reported that they perceived that the viewing took place during dignified circumstances, which suggests that complicating factors like an unprepared or damaged body might be accepted if the bereaved are carefully informed and supported during the viewing.

Our finding that the majority of the persons that did not view the body did not wish they had, agrees with previous findings [1], [2]. There are also some who did not view who afterwards wish that they had. Chapple and Ziebland [1] showed that some respondents changed their mind regarding what they thought was best for them and that some, afterwards, were ambivalent about whether their decision was the best one. One explanation might be that these individuals may hold a belief that viewing is necessary for a healthy recovery, a view suggested by some respondents in our study as well as in the grief literature. Dublin and Sarnoff's_ENREF_2_ENREF_2 review [2] from 1986 concludes that bereaved persons should be offered the opportunity to view the body but also stress that those who are reluctant or unwilling to do so must be supported by being told that their decision was the right one for them.

Our hypothesis that those who viewed the body in a formal setting would have lower levels of psychological morbidity than those who did not view was not supported by our findings. In contrast, viewing was associated with a higher risk of reliving the child's death through nightmares and intrusive memories, although no differences could be found regarding anxiety, depression or avoidance two to five years after the death (Table 4). Research on the psychological effects of viewing the body after a suicide loss is sparse. We found two studies that explored how confronting the body (at the scene of the death and at a formal setting) affected the level of grief difficulties among suicide-bereaved relatives [18], [19]. In this paper we chose to restrict the discussion to findings concerning viewing in the formal setting. Callahan's study [18] included 210 persons who had lost a family member or a close friend to suicide. The bereaved were all participants in suicide support groups and data were collected in Michigan (1989 to 1993) and Chicago (1995 to 1996) with the average elapsed time since loss being four years. Callahan hypothesized that “Not seeing the deceased's body at the funeral or memorial service” was associated with higher levels of grief as measured by the Grief Experience Questionnaire but found no impact on the overall level of grief. Feigelman and co-workers_ENREF_12 [19] studied a sub-group of 462 parents who had lost their son or daughter to suicide during a time span of less than a year to more than 10 years. An abbreviated version of the Grief Experience Questionnaire was used for the outcome measures and the parents were identified by suicide support groups in the USA. Feigelman and co-workers hypothesized that the suicide-bereaved who had viewed the body prior to the burial or cremation (n = 189) would experience higher levels of grief difficulties than those who had not viewed the body prior to the burial or cremation (n = 96) (the parents that had seen the body at the site of the death were not included in any of the groups). Feigelman and co-workers found that those who had not viewed had a lower level of grief difficulties than those who had viewed. Our findings on the psychological effect of viewing the body in a formal setting are in line with Callahan and Feigelmans's findings, thus challenging the notion that viewing the body is necessary for a healthy grief recovery.

Our study has several strengths; one is the large sample of suicide-bereaved parents, all identified through nationwide high-quality registers. Another is the high participation rate among both men and women. Some individuals were parents of the same child but all were contacted separately and received their own questionnaire. Some parents may have discussed their answers with the other parent, but our experiences from the in-depth interviews and the validation interviews were that the parents primarily described their own experiences and that the mother and fathers experiences often differed from each other's. Our study also has limitations. The opportunity and decision to view or not to view the body are influenced by numerous factors, some of them known, others not. We have no quantitative data on whether the parents wanted and/or had the choice to view the body at the time of death. However, the written comments to the questions suggest similar to previous studies that the decision often was influenced by other persons and circumstances surrounding the body [1], [5]. Ideally additional data concerning “viewing the body” would include information from different sources (observations and self-rated), immediate reactions and a range of mental health outcomes measured at different times of follow-up. However, due to methodological issues this was not possible. For example, collecting longitudinal data and adding more question would most likely have compromised the response rate and thereby the validity of the study. For similar reasons we also lack information about possible confounders' related to different personality and coping strategies, since existing inventories were considered too immense and the study-specific questions from the preparatory study imprecise [9]. Data was collected retrospectively. Thus some of the answers may be affected by recall-induced problems such as issues concerning whether the parents perceived that the viewing of their child took place under dignified circumstances. However, most of our outcomes concern how the parents feel today or how they have felt during the last month or the last two weeks and thus are not influenced by memory-bias. Some answers might have been affected by defence mechanisms, e.g. a too painful memory could be suppressed or replaced by a less painful one. During the in-depth-interviews and the validation interviews, however, we did not find that the parents had difficulties in answering questions regarding sensitive issues such as where they saw the body of their dead child. We therefore consider the recall-induced problems to have a minor if any effect on the effect measures presented in this paper.

We addressed the threats to validity by employing epidemiological methods as transferred to this field by the hierarchical step-model for study design, analysis and data interpretation [20]. Efforts to reduce the problem of misclassification included a thorough preparatory study, developing and testing the questions and the psychometric scales in close collaboration with parents from the study-population [9]. When choosing scales we considered psychometric properties, relevance to our research questions and whether the format of the scale was suitable for our questionnaire. We chose the psychometrically tested scales PHQ-9 and GAD-2 because they have been used and tested in similar study-populations and have shown high reliability and validity despite of their compactness [11], [14]. We used psychometrically validated measures when possible. However, since most concepts in this study have not previously been studied we had to design ad hoc questions that we validated through a comprehensive preparatory study [6], [9].

Our main outcomes were measured by psychometric as well as study-specific questions and we have no reason to believe that the ones who viewed the body and the ones that did not view differ systematically in their response to these questions [21]. It is likely that the fundamental manifestations of grief are universal but still, generalisation to other populations may be compromised by culture-specific issues. For ethical and methodological reasons we could only include Swedish speaking parents in this survey. However, some of our findings might be as trustworthy in other populations and settings. We have therefore described our research process and data in detail so that other researchers and clinicians can decide whether our findings are applicable for them in their settings [6], [9].

In summary, in this Swedish population-based study, we found that by and large everyone that had viewed their deceased child in a formal setting did not regret doing so. We also found that the majority of the parents that did not view their deceased child did not wish that they had. We found no support for the position that viewing the body in a formal setting had a positive effect on the psychological outcomes, two to five years after the loss. This is the first larger population-based survey on the subject and the study needs to be repeated in other settings and study populations ideally using longitudinal design. More qualitative and observational studies are also warranted to capture the complexity of the subject. Although no recommendations can be made, our findings suggest that it is the bereaved person that should make the decision to view or not to view the body and that the officials may support the parents in their decision by carefully informing about the child's appearance and how the viewing may be altered, for example, by shielding parts of the body. Facilitating mental preparation and a thoughtful caretaking may also reduce the significant minority of parents that reported that they regretted the viewing or that they perceived the viewing as less than dignified. For parents that seek advice, the officials may also tell them that previous research suggests that most parents that want to see their child do not regret doing so and that viewing often is perceived as helpful although not necessary for a healthy recovery.

## Supporting Information

Table S1 Hypotheses supplement.
(DOCX)
