# Supplementary material for: Viewing the Body after Bereavement Due to Suicide: A Population-Based Survey in Sweden
Source: PLoS One. 2014 Jul 7;9(7):e101799. doi: 10.1371/journal.pone.0101799 (PMC4085007; doi:10.1371/journal.pone.0101799)
Supplement: Table S1 — Hypotheses supplement. (DOCX) [file pone.0101799.s001.docx]

|  | **Regret formal** | **Nightmares** | **Intrusive** | **Avoiding** | **Avoiding** | **Depression** | **Anxiety** |
| --- | --- | --- | --- | --- | --- | --- | --- |
| No/total no (%) | **viewing^*^** |  | **memories** | **thoughts** | **places** | **(PHQ-9)** | **(GAD-2)** |
| **Saw^*†^ the dead child** |  |  |  |  |  |  |  |
|  | n=460 | n=666 | n=666 | n=666 | n=666 | n=666 | n=666 |
| No | - | 29/171 (17.0) | 96/170 (56.5) | 50/170 (29.4) | 44/167 (26.3) | 26/169 (15.4) | 35/170 (20.6) |
| Yes | 16/446 (3.6) | 117/493 (23.7) | 312/487 (64.1) | 163/490 (33.3) | 127/489 (26.0) | 89/484 (18.4) | 104/486 (21.4) |
| RR (CI 95%) | - | 0.71 (0.49 to 1.03) | 0.88 (0.76 to 1.02) | 0.88 (0.68 to 1.15) | 1.01 (0.76 to 1.36) | 0.84 (0.56 to 1.25) | 0.96 (0.68 to 1.35) |
| Trend test P value | **-** | **0.048** | **0.037** | 0.485 | 0.960 | **0.009** | 0.823 |
| **Discovered/saw body** |  |  |  |  |  |  |  |
| **at the site of death** | n=460 | n=666 | n=666 | n=666 | n=666 | n=666 | n=666 |
| Yes | 5/111 (4.5) | 31/147 (21.1) | 88/145 (60.7) | 46/145 (31.7) | 35/144 (24.3) | 24/143 (16.8) | 29/145 (20.0) |
| No | 11/333 (3.3) | 114/512 (22.3) | 317/507 (62.5) | 167/510 (32.7) | 136/507 (26.8) | 90/505 (17.8) | 109/506 (21.5) |
| RR (CI 95%) | 1.36 (0.48 to 3.84) | 0.95 (0.67 to 1.35) | 0.97 (0.84 to 1.13) | 0.97 (0.74 to 1.27) | 0.91 (0.66 to 1.25) | 0.94 (0.62 to 1.42) | 0.93 (0.64 to 1.34) |
| Trend test P value | 0.537 | 0.833 | 0.613 | 0.863 | 0.627 | 0.963 | 0.637 |
| **Viewed the body** |  |  |  |  |  |  |  |
| **in a formal setting^*^** | n=460 | n=666 | n=666 | n=666 | n=666 | n=666 | n=666 |
| No | Not applicable | 31/202 (15.3) | 109/200 (54.5) | 57/200 (28.5) | 52/197 (26.4) | 30/199 (15.1) | 42/200 (21.0) |
| Yes | 16/446 (3.6) | 114/460 (24.8) | 297/455 (65.3) | 156/458 (34.1) | 118/457 (25.8) | 85/452 (18.8) | 97/454 (21.4) |
| RR (CI 95%) | Not applicable | **0.62 (0.43 to 0.89)** | **0.84 (0.72 to 0.96)** | 0.84 (0.65 to 1.08) | 1.02 (0.77 to 1.35) | 0.80 (0.55 to 1.17) | 0.98 (0.71 to 1.36) |
| Trend test P value |  | **0.005** | **0.007** | 0.276 | 0.927 | **0.005** | 0.893 |
| **Perceived formal** |  |  |  |  |  |  |  |
| **viewing worthy** | n=460 | n=460 | n=460 | n=460 | n=460 | n=460 | n=460 |
| No | 5/18 (27.8) | 7/19 (36.8) | 14/18 (77.8) | 10/19 (52.6) | 10/19 (52.6) | 3/18 (16.7) | 1/18 (5.6) |
| Yes | 11/423 (2.6) | 106/436 (24.3) | 279/432 (64.6) | 144/434 (33.2) | 106/433 (24.5) | 81/429 (18.9) | 96/431 (22.3) |
| RR (CI 95%) | **10.68 (4.15 to 27.51)** | 1.52 (0.82 to 2.79) | 1.20 (0.93 to 1.56) | **1.59 (1.02 to 2.48)** | **2.15 (1.36 to 3.40)** | 0.88 (0.31 to 2.53) | 0.25 (0.04 to 1.69) |
| Trend test P value | **<0.001** | 0.284 | 0.073 | 0.056 | **0.005** | 0.236 | 0.114 |
| **Circumstances** |  |  |  |  |  |  |  |
| **related to the suicide** | n=460 | n=666 | n=666 | n=666 | n=666 | n=666 | n=666 |
| Violent suicide^‡^ | 8/375 (2.1) | 120/549 (21.9) | 340/543 (62.6) | 175/545 (32.1) | 146/541 (27.0) | 92/540 (17.0) | 111/541 (20.5) |
| Poisoning^§^ | 7/61 (11.5) | 21/101 (20.8) | 59/100 (59.0) | 36/101 (35.6) | 21/101 (20.8) | 22/99 (22.2) | 25/101 (24.8) |
| RR (CI 95%) | **0.19 (0.07 to 0.49)** | 1.05 (0.70 to 1.59) | 1.06 (0.89 to 1.27) | 0.90 (0.68 to 1.20) | 1.30 (0.87 to 1.95) | 0.77 (0.51 to 1.16) | 0.83 (0.57 to 1.21) |
| Trend test P value | **<0.001** | 0.895 | 0.625 | 0.649 | 0.150 | 0.856 | 0.339 |
| Death notice ≥ 24h | 3/55 (5.5) | 19/100 (19.0) | 54/100 (54.0) | 26/100 (28.0) | 24/99 (24.2) | 17/98 (17.3) | 21/99 (21.2) |
| Death notice < 24h | 13/385 (3.4) | 125/557 (22.4) | 347/550 (63.1) | 182/553 (32.9) | 144/550 (26.2) | 97/548 (17.7) | 116/550 (21.1) |
| RR (CI 95%) | 1.61(0.48 to 5.49) | 0.85 (0.55 to 1.31) | 0.87 (0.71 to 1.04) | 0.85 (0.61 to 1.19) | 0.93 (0.64 to 1.35) | 0.98 (0.61 to 1.57) | 1.01 (0.67 to 1.52) |
| Trend test P value | 0.445 | 0.456 | 0.216 | 0.334 | 0591 | 0.607 | 0.943 |

^*^ Parents stated that viewed their dead child in a formal setting: “Emergency department or ward”, “Hospital church”, “Department of forensic medicine”, and “Funeral home”. ^†^Parents that found their dead child, were present at the time of death and, witnessed the suicide. ^‡^ Parents that stated that their child committed suicide by “Hanging, strangulation, suffocation”, “Drowning”, “By moving vehicles, “Jumping from a height”, “By firearm discharge”, “Cutting or stabbing” and, “By fire.^§^ Formulated in the questionnaire as “Poisoning for example by medication, chemicals or some kind of gas”.
